# A novel integrated angioscope-laser system for atherosclerotic carotid artery occlusion: Feasibility and techniques

**DOI:** 10.3389/fsurg.2022.937492

**Published:** 2022-10-10

**Authors:** Boqian Zhang, Guiyun Zhang

**Affiliations:** ^1^ Shanghai Jiao Tong University School of Medicine, Shanghai, China; ^2^ Department of Neurovasclar Intervention and Neurosurgery, Shanghai General Hospital, Shanghai Jiao Tong University School of Medicine, Shanghai, China

**Keywords:** atherosclerotic carotid artery occlusion, angioscope, laser ablation, plaque, stenosis

## Abstract

**Introduction:**

Atherosclerotic extracranial carotid artery stenosis accounts for about 20%–30% of all strokes, which is one of the leading causes of adult morbidity and mortality. Although carotid endarterectomy (CEA) is still the mainly operational manner for atherosclerotic carotid artery stenosis/occlusion (ACAS/ACAO), and carotid angioplasty and stenting (CAS) have been used as an alternative, both CEA and CAS have limitations of their own, such as extensive invasiveness and in-stent restenosis.

**Methods:**

In this study we established a novel interventional system *in vitro* to take advantage of both CEA and CAS. Twenty consecutive carotid atherosclerotic plaques were harvested from the patients who underwent CEA. The plaques were randomized into two groups and inserted into the pruned and sutured descending aortas of the swine *in vitro*. The ZebraScope™ was modified with a protective device on its flexible tip, so that the plaque could be dissected from the wall of parent carotid artery and ablated completely without damage to the carotid artery. The holmium:YAG (Ho:YAG) and thulium fiber laser (TFL) generators were alternately used when needed.

**Results:**

All the carotid atherosclerotic plaques were completely ablated by Ho:YAG laser and/or TFL. The Ho:YAG laser was more effective for the atherosclerotic plaques with severe calcification, while the TFL was more suitable for those with moderate calcification. There were still some thermal injury spots on the inner wall of the parent carotid artery caused by the laser in the non-protected group B. In the protected group A, on the contrary, there was no even a thermal injury spot was found on the relevant location except for one sample. The difference of ablating duration was statistically significant between group A (36.5 ± 4.79 min) and group B (63.4 ± 6.55 min) (*P* < 0.01).

**Conclusion:**

According to our knowledge, this is the first attempt to ablate carotid atherosclerotic plaques assisted by the ZebraScope™ *in vitro*. The protective and dissecting device on the tip of the angioscope makes it safe and visible when the ablation is performed to carotid atherosclerotic plaques. The Ho:YAG laser and TFL are effective and safe for ablating the plaque *in vitro*.

## Introduction

Large vascular occlusion or stenosis, including extra and intracranial large artery, is a very common cause of ischemic stroke. It accounts for about 4%–46.6% of ischemic strokes in population-based studies from the west to the east (1–10). Extracranial internal carotid artery stenosis, especially the ACAS/ACAO, is the most leading cause of large artery stroke accounting for about 20%–30% of all ischemic strokes (11–13). CEA, as the golden standard treatment for carotid stenosis, distinctly reduces the risk of recurrent stroke of patients with symptoms such as stroke or transient ischemic attack (14). CAS has been developed as an alternative to CEA for treating carotid stenosis without necessitating surgery. Both CEA and CAS are equally effective in preventing recurrent stroke in the first 4-year following procedures (15), but recurrent stenosis, compared with CEA, is found to be more common after CAS. On the other hand the risks of peri-procedural myocardial infarction, cranial nerve palsy and access site hematoma caused by CEA should not be neglected (16). The best treatment approach for patients with symptomatic carotid stenosis (occlusion) remains to be determined in the long term. To grope for a new approach for ACAS/ACAO is what we concern about. In this study *in vitro*, we established a model to simulate the conditions of ACAO and verified the efficacy and safety of the novel integrated angioscope-laser system.

## Materials and methods

### The modified ZebraScope™

**Figure 1:**
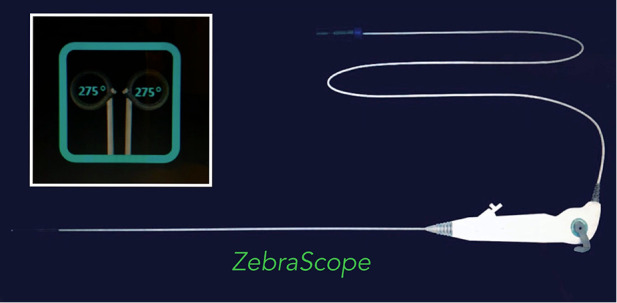
Zebrascope was composed by scope handle, scope body and data wire. There was an entrance on the handle through which the laser fiber was introduced into the scope body. During the procedure, the surgeon could rotate the handle and curve the flexible tip of the scope body up and down through the controller on the handle.

**Figure 2:**
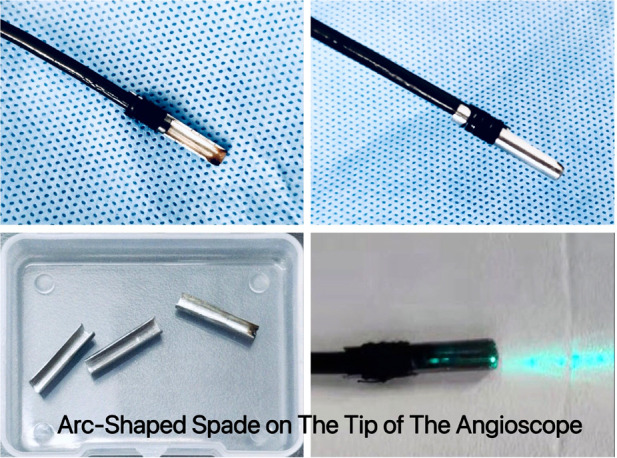
Arc-shaped spade was fixed on the tip of the angioscope. The spade was made by stainless steel with a radius angle of 180–220° (0.1 mm thickness). The arc-shaped spade had two kinds of functions, one was for dissecting the plaque from the wall of internal carotid artery by rotating and pushing the scope handle, the other was to be used as a physical barrier to protect the artery wall from being injured by the lasers.

**Figure 3:**
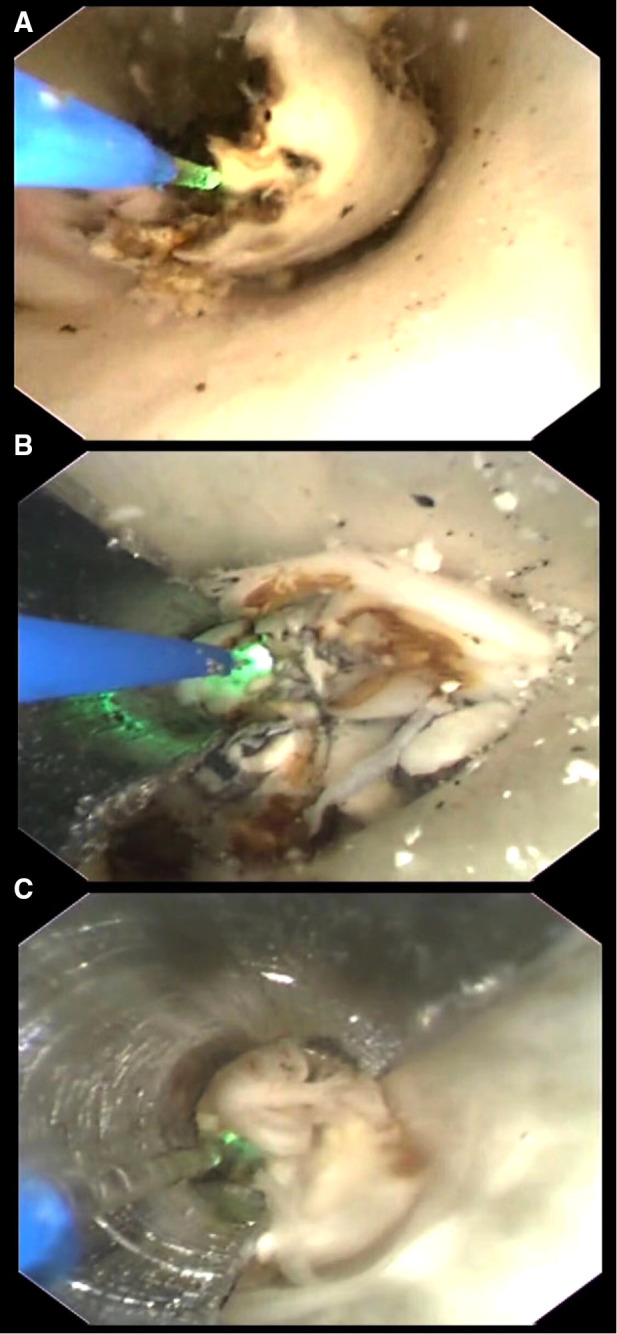
(**A**) Ablating without protective device. Procedure under this condition usually caused injury to the wall as many thermal injury spots were seen with naked eyes. (**B**) Ablating under the protection of a spade with a radius angle of 180°. To get a good protection to the artery wall, the spade with a radius angle of 180° was firstly used with the optical fiber in a diameter of 200 μm. (**C**) Ablating under the protection of a spade with a radius angle of 220°. To get a good protection to the artery wall, the spade with a radius angle of 220° was firstly used with the optical fiber in a diameter of 165 μm.

ZebraScope™ (Anhui Happiness Workshop Medical Equipment Co.; Ltd, China) was a single-use flexible ureteroscope which was initially used for the examination or treatment of calculuses in the ureter and renal pelvis (Figure 1). In this study we used it as an angioscope. The ZebraScope™ was composed mainly of angioscope body and imaging host (Version: XFGC-UZ-A). The angioscope had a flexible distal segment with the length of 5 cm, which could be curved up or down even to 275° with a smallest radius of 8 mm in the same plane by the controller on the handle. The coaxial design for the canals of imaging data wire and fiber (shared with the irrigation canal) made the whole body of the scope to twist with a 1 : 1 torque. The Complementary Metal Oxide Semiconductor (CMOS) on the tip of the scope with a diameter of 7.4 F and a high definition of 160,000 pixels, assisted with a sustainable normal saline irrigation at a flow of 50 ml/min, made it easy to operate in the carotid artery and gave us a clear field of view. To prevent the media of the carotid artery from being damaged by the laser, we designed an arc-shaped spade made by stainless steel with a radius angle of 180–220° (0.1 mm thickness) and fixed it on the tip of the angioscope (Figure 2). The fiber tip should be kept about 0.5 mm proximally to the anterior edge of the spade, so that the plaque could be dissected step by step from the wall of carotid artery by the spade and ablated by laser from the fiber (Figure 3).

### The Laser systems and fibers

**Figure 4:**
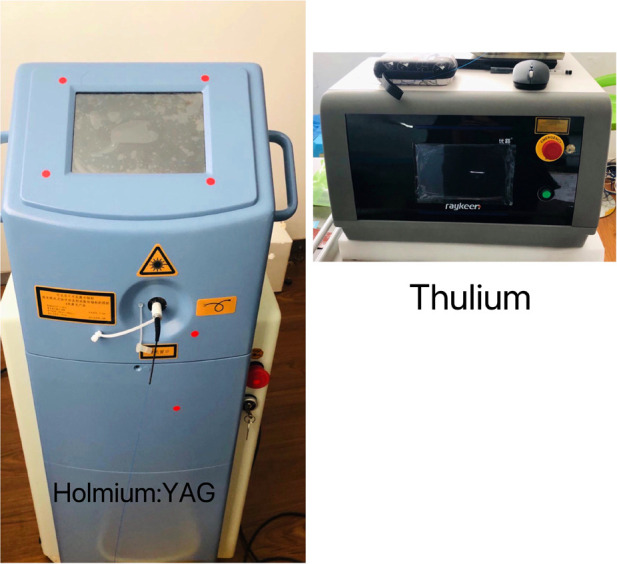
Laser generator of holmium:YAG and thulium. Usually, the body of laser generator of Holmium:YAG was larger than that of Thulium. And the latter seemed more effective in the plaques without severe calcification.

The Ho:YAG laser generator (Version: SRM-H1B) with a wavelength of 2120 nm and the super pulsed TFL generator (Version: SRM-T1F) with a wavelength of 1940 nm were provided by Raykeen Laser Technique Co, Ltd, Shanghai, China (Figure 4). Single-use laser fibers with core diameters of 165 µm and 200 µm were used for TFL and Ho:YAG laser respectively.

### Artificial atherosclerotic carotid artery occlusion

**Figure 5:**
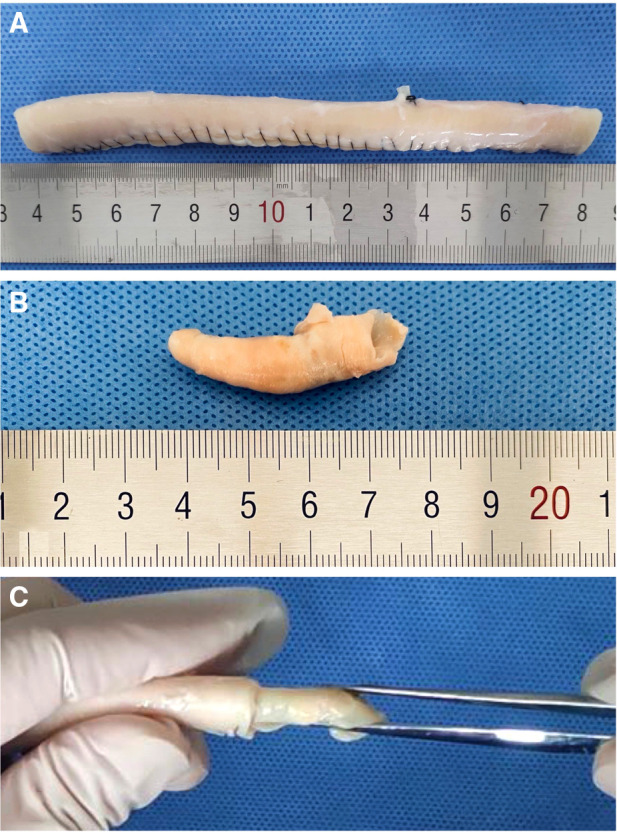
(**A**) Carotid artery made with a pruned and sutured descending aorta. (**B**) Atherosclerotic carotid artery plaque from the patient. It was confirmed as a stable plaque by ultrasonography. (**C**) We inserted the plaque into the “carotid artery” to make an atherosclerotic carotid artery occlusion state.

Twenty consecutive carotid atherosclerotic plaques were harvested from the patients who underwent CEA. Ultrasound and computed tomography angiography (CTA) were used to evaluate the characters and calcification degree of the plaques before CEA, The plaques' characters were confirmed by ultrasound (stable vs. vulnerable = 8:12); Ultrasonographic criteria of stable plaque was defined as that with thicker and holonomic fiber cap, little lipid core, while the plaque with thinner or incomplete fiber cap, large lipid core, ulceration, vasa vasorum intraplaque confirmed by contrast-enhanced ultrasound was classified as the vulnerable. CTA not only showed the stenosis but also the calcification degree, the length of calcification <1/4 perimeter of internal carotid artery was recorded as “+”, that between 1/4 and 1/2 perimeter was recorded as “++”, and that >1/2 perimeter was recorded as “+++”. Having been soaked in the formalin for at least one week and recorded with their volume measured by drainage method, the plaques were randomized into two groups (A/B, with/without protective device on the tip of the angioscope) and inserted into the pruned and sutured descending aortas of the swine *in vitro* (Figure 5). These artificial carotid arteries carrying atherosclerotic plaques were used to simulate the conditions of atherosclerotic carotid artery occlusion or severe stenosis of the patients.

### Experimental procedure

The descending aortas of the swine in the −80°C refrigerator were taken out and put into the cold water for a 5-minute thawing. After being thawed, they were pruned and sutured into the carotid arteries with an inner diameter of 5 mm and a length of 15 cm. The volume of each plaque measured by drainage method was recorded, and then the plaque was inserted into the artificial carotid artery. In this way the artificial carotid arteries carrying atherosclerotic plaques were made and put into the test cubes in the ice-cold normal sodium for laser ablating. When the operation started, the irrigational flow was controlled as 50 ml/min by the peristaltic pump. The irrigational saline from the 3-litre-bag was kept at an ordinary temperature. All the plaques were ablated completely. The volume of ablated plaque tissue in each sample ranged from 0.38 to 0.58 ml, which was list on Table 1. The size of the debris was 50–100 µm. All the debris could be rinsed out by the irrigating saline in this study *in vitro*. When we apply this system to the patient in the real world, a balloon catheter system was used (12–14 F balloon guiding catheter (BGC), as the first one, was used for delivering the integrated angioscope-laser system and occluding the proximal CCA; the second (4 F) and the third (2 F) ones were used to occlude the ICA distal to the plaque and the origin of ECA respectively). With the inflating of balloon catheter system, the plaque would be immerged in the normal saline with ordinary temperature without any blood. The saline would not be irrigated into the brain, it would be drained through the gap between the first balloon catheter and the integrated angioscope-laser system. To avoid the ipsilateral ischemia, the second balloon catheter was designed with two cavities, one was for inflating the balloon, and the other was for the delivering of blood from the patient's femoral artery into the ipsilateral hemisphere. The volume of each plaque, duration of ablation for each plaque, thermal injury spots on the inner wall of the carotid artery, calcification degree, and characters of plaques (vulnerable/stable) were recorded down and listed on Table 1.

**Table 1:** Volume of plaques, duration of ablation, thermal injury spots, calcification degree, and characters of plaques in Group A and B.

No.	Group A					Group B				
	Vol-pla (ml)	Dur-abl (min)	The-spo (number)	cal-deg (−/+)	V/S-plaque	Vol-pla (ml)	Dur-abl (min)	The-spo (number)	Cal-deg (−/+)	V/S-plaque
1	0.39	30	0	+	V	0.42	60	12	+	V
2	0.42	35	0	++	V	0.53	70	15	++	S
3	0.58	45	1	+++	S	0.50	58	9	+	V
4	0.45	38	0	+	V	0.38	65	12	++	V
5	0.56	40	0	++	S	0.49	70	8	+	V
6	0.40	33	0	+	V	0.37	52	13	++	S
7	0.38	30	0	+	V	0.51	60	15	++	S
8	0.41	35	0	+	V	0.40	68	10	+	V
9	0.50	39	0	++	S	0.47	59	11	+	V
10	0.49	40	0	++	S	0.51	72	16	++	S

Vol-pla (ml), volume of the plaque (ml); Dur-abl (min), duration of ablation (min); The-spo (number), number of thermal injury spots; Cal (−/+), calcification degree; “−”, negative; “+”, slight calcification; “++”, moderate calcification; “+++”, severe calcification; V/S-plaque, vulnerable or stable plaque; “V”, vulnerable plaque; “S”, stable plaque.

### Statistical analyses

Data were analyzed by using post-hoc test of variance (ANOVA) by the IBM SPSS Software (version 26.0). Data results were showed as Mean ± SD. A *p*-value of 0.05 or less was considered significant.

## Results

**Figure 6:**
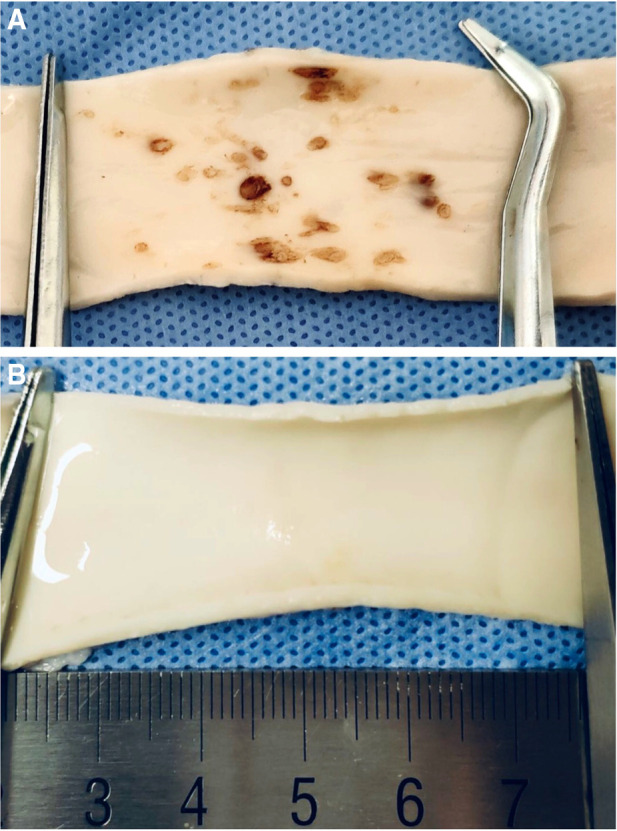
(**A**) Many thermal injury spots were found in the inner wall of the “carotid artery” after the ablating of the plaque without protection. (**B**) No thermal injury spots were found in the inner wall of the “carotid artery” after the ablating of the plaque with spade-protection.

The difference of the volume (group A, 0.46 ± 0.07 ml; group B, 0.46 ± 0.06 ml) and calcification degree of the plaques between group A and B was not statistically significant (*P* > 0.05). The difference of ablating duration was statistically significant between group A (36.5 ± 4.79 min) and group B (63.4 ± 6.55 min) (*P* < 0.01). The difference of number of thermal injury spots was statistically significant between the two groups (*P* < 0.01) (Figure 6).

## Discussion

CEA and CAS have emerged in succession as the effective measures for the prevention of stroke in patients with symptomatic and asymptomatic severe carotid artery stenosis (15, 17, 18). To minimize the damage to the intima or media is the soul of CEA. Any other operation modality, as a non-inferior or superior alternative to CEA or CAS, must have this characteristic first. The second characteristic of an ideal operation modality for ACAO/ACAS must be minimal invasive to the patients. The third must be less of durations and convenient for popularizing. The forth must keep a normal perfusion to the brain during the procedures and the fifth must have lower rate of embolic and restenosis. As a matter of fact, both CEA and CAS still have the limitations of their own, such as extensive invasiveness and in-stent restenosis.

Laser angioplasty, accompanied by enthusiastic expectations, emerged as an endovascular technique for peripheral arterial revascularization at the end of 1980s. It was very appealing that laser had the potential to ablate atherosclerotic plaque and restore the normal anatomy through minimal invasive approach. From the end of 1980s to 1990s, there were a lot of researches about the safety and feasibility of using laser for ablating atherosclerotic plaque, as well as the unique feature of different kind of laser (19,20). However, the enthusiasm declined as the fact of lower long-term patency rates after initial procedures (21). As a true step forward in the endovascular treatment of occlusive disease (22), excimer laser angioplasty nowadays is commonly used in the field of peripheral artery disease (atherosclerotic plaques) and has shown a satisfied outcome with a mean follow-up of 15 ± 7 months. The primary patency was 65% ± 20% and the mean distal embolism rate during the procedure was 5% (23). But excimer laser alone couldn't finish the angioplasty without balloons, and for a chronic white fibrous thrombus the excimer laser was not effective. With the problem of embolic and punctured injury, utility of laser in coronary artery occlusion was also limited. We thought that these unsatisfied results were caused by inappropriate kinds of lasers and incomplete ablating. The laser could only remove partially or totally the plaque itself, but with the thickened intima left on the media. Laser alone could not get the same results at an anatomical level as that of CEA. So we can't use this modality for ACAO or ACAS directly.

Ho:YAG laser, as the first candidate for lithotripsy because of its property to fragment all stone compositions, efficiencies and safety profiles, has been used for three decades (24,25). It operates at 2,120 nm in pulsed mode and is highly absorbed in liquid water. In pulsed mode, the sudden formation of a vapor bubble after emission could make calculus and hard tissue broken into pieces. This interaction with water also makes it safe with an optical penetration depth limited to 400 μm. As the latter, the Super Pulsed TFL comes with potential advantages over Ho:YAG laser such as higher ablation volumes during lithotripsy and production of thinner particles (26–30). For TFL, it has been optimized to emit at a wavelength of 1,940 nm, which is closely matching the near-infrared absorption peak of liquid water at 22°C (31). Because the absorption coefficient of the TFL is more than four-fold higher than Ho:YAG, a lower threshold and higher ablation efficiency can be expected in favor of the TFL at equivalent pulse energies.

To confirm the safety and efficacy of laser for ablating carotid atherosclerotic plaque is what we concern most. Even to this day there are still some primary problems which limit the utility of laser in ACAO or ACAS. As we know this kind of procedures are performed under digital subtraction angiography (DSA) and without distal protection. It is an unvisualyzed operation which doesn't provide a clear field for dissecting atherosclerotic plaque from the media like that of CEA. With the five characteristics mentioned above in our minds we took the first step with the novel integrated angioscope-laser system for ACAO. This system had an integrated feature of minimal invasiveness, visualization and anatomical dissection plus laser ablation.

In this study, for different type of plaque, there were some different effects between these two kinds of lasers. we used Ho:YAG laser for those atherosclerotic plaques with severe calcification. It seemed that the TFL was more efficient for those with moderate calcification, especially for the thickened intima. This different performance to calcification might be caused by the limitation of lower power with 8–12 W. The high performance of TFL might not be shown here. With the elevating of power and frequency more and more bubbles emerged in the operation field suggesting a great jump of temperature. The irrigating flow had to be increased to get an optimal cooling environment.

Both the 180° and 220°-arc-shaped spade were effective when used with the fibers with a diameter of 200 µm and 165 µm respectively. As the limitation of the Ho:YAG laser itself, optical fibers with a core diameter of 200 μm or larger was needed (32).

Although the fiber with a smaller diameter, usually used with TFL, brought an enough irrigating flow for cooling and washing, it caused a comparative large gap in the canal of the angioscope. Because of the larger gap, an arc-shaped spade in a larger radius angle was needed in case of injury to the carotid artery. With the help of the arc-shaped spade, the atherosclerotic plaques could be dissected from the wall of parent carotid artery without any thermal injuries caused by the laser except for one plaque with severe calcification in the protected group. The only thermal injury spot was probably caused by mistake of the operator as longer procedure duration. The durations of protected group were significantly shortened compared with those in non-protected group. We attributed the difference to that the operator could precede more confidently and fast with the help of the arc-shaped spade on the tip of the angioscope.

## Limitations

Some limitations to this study should be noticed. First, the two kinds of lasers were used for the preselected cases by virtue of the degree of calcification of the plaque, it was not used randomly. the second, the model of ACAO was not completely in accord with the actual. For the *in situ* atherosclerotic plaque, the contents of lipid core and calcified tissues usually located within the thickened intima and covered by complete or incomplete fiber cap. To separate the plaque from the media of carotid artery we should start from an incision of the intima. This was the difference between our model and the plaque *in situ*. Another limitation was that the ablation was not carried out in an ongoing closed circulation environment. We couldn’t verify the embolic incidence as in the closed. The last, the carotid arteries were not for light microscopy because of intact of carotid artery in the protected group. Further study should be founded in a closed unidirectional circulation canal with our ACAO or ACAS model inserted. At the same time, a temporary balloon occlusion with artery-catheter-artery-bypass should be established when this utility is going to be verified in the corpses. Both angioscopy and laser atherectomy are well established techniques, although the minimal invasive and real time operation under the integrated angioscope-laser system without occluding the blood flow to brain is amazing, there is still a long way to go for the utility of this integrated angioscope-laser system to the patients in the real world. we must establish a balloon catheter system, as illustrated in the part of “Experimental Procedure”. What's more, a more effective and safe operative related accessories should be innovated as a supplement. Such as a double cavity balloon catheter with more flexible part in the distal and more supportable part in the proximal, an angioscope with high definition of 4 K (3480 × 2,160 pixels) will give us more details and make the operation more precise and safer. With all the verifications above and devices innovated, our novel integrated angioscope-laser system might be an alternative for ACAO or ACAS.

## Conclusion

We have designed a novel integrated angioscope-laser system for ACAO or ACAS and verified the efficacy and safety of arc-shaped spade, Ho:YAG laser and TFL. According to our knowledge, this is the first attempt to ablate carotid atherosclerotic plaques assisted by the ZebraScope™ *in vitro*. The protective and dissecting device (arc-shaped spade) on the tip of the angioscope, designed by the author, makes it safe and visible when the ablation is performed to the carotid atherosclerotic plaques. The Ho:YAG laser and TFL, used alternately, are effective and enough for the carotid atherosclerotic plaques *in vitro*.

## Data Availability

The original contributions presented in the study are included in the article/Supplementary Material, further inquiries can be directed to the corresponding author/s.
